# Myoelectric activity during electromagnetic resistance alone and in combination with variable resistance or eccentric overload

**DOI:** 10.1038/s41598-023-35424-w

**Published:** 2023-05-22

**Authors:** Hugo Zambrano, Xavier Torres, Max Coleman, Martino V. Franchi, James P. Fisher, Douglas Oberlin, Bas Van Hooren, Paul A. Swinton, Brad J. Schoenfeld

**Affiliations:** 1 Department of Exercise Science and Recreation, CUNY Lehman College, Bronx, NY USA; 2 Department of Biomedical Sciences, University of Padova, Padua, Italy; 3 Department of Sport and Health, Solent University, Southampton, UK; 4 Department of Nutrition and Movement Sciences, Maastricht University Medical Centre+, 6229 HX Maastricht, The Netherlands; 5 School of Health Sciences, Robert Gordon University, Aberdeen, UK

**Keywords:** Physiology, Anatomy

## Abstract

The purpose of this study was to compare the effects of electromagnetic resistance alone, as well as in combination with variable resistance or accentuated eccentric methods, with traditional dynamic constant external resistance exercise on myoelectric activity during elbow flexion. The study employed a within-participant randomized, cross-over design whereby 16 young, resistance-trained male and female volunteers performed elbow flexion exercise under each of the following conditions: using a dumbbell (DB); using a commercial electromagnetic resistance device (ELECTRO); variable resistance (VR) using a setting on the device that attempts to match the level of resistance to the human strength curve, and; eccentric overload (EO) using a setting on the device that increases the load by 50% on the eccentric portion of each repetition. Surface electromyography (sEMG) was obtained for the biceps brachii, brachioradialis and anterior deltoid on each of the conditions. Participants performed the conditions at their predetermined 10 repetition maximum. " The order of performance for the conditions was counterbalanced, with trials separated by a 10-min recovery period. The sEMG was synced to a motion capture system to assess sEMG amplitude at elbow joint angles of 30°, 50°, 70°, 90°, 110°, with amplitude normalized to the maximal activation. The anterior deltoid showed the largest differences in amplitude between conditions, where median estimates indicated greater concentric sEMG amplitude (~ 7–10%) with EO, ELECTRO and VR compared with DB. Concentric biceps brachii sEMG amplitude was similar between conditions. In contrast, results indicated a greater eccentric amplitude with DB compared to ELECTRO and VR, but unlikely to exceed a 5% difference. Data indicated a greater concentric and eccentric brachioradialis sEMG amplitude with DB compared to all other conditions, but differences were unlikely to exceed 5%. The electromagnetic device tended to produce greater amplitudes in the anterior deltoid, while DB tended to produce greater amplitudes in the brachioradialis; amplitude for the biceps brachii was relatively similar between conditions. Overall, any observed differences were relatively modest, equating to magnitudes of ~ 5% and not likely greater than 10%. These differences would seem to be of minimal practical significance.

## Introduction

Resistance training (RT) promotes a plethora of health- and functional-related benefits including improvements in muscle strength, power, hypertrophy, and various cardiometabolic markers, among others^1,2^. Typical RT programs involve performing a series of repetitions with dynamic constant external resistance (DCER), whereby the external load remains constant throughout performance of both concentric and eccentric muscle actions^3^. DCER can be accomplished via the use of free weights and various machines. Electromagnetic technology, which creates resistance via opposing magnetic fields, also has been employed in this regard^4^, although its use in commercial and research settings is limited to date.

RT programs must incorporate the principle of progressive overload to elicit continued adaptations over time. Simply stated, progressive overload involves successively placing greater than normal demands on the exercising musculature^5^. This is accomplished via the manipulation of RT variables, which can be achieved in myriad ways. A variety of advanced training methods have been proposed to facilitate progressive overload, particularly in more experienced lifters^6^. Two of these methods, eccentric overload (EO) and variable resistance (VR), modify DCER in an attempt to increase loading capacity during performance. Conceivably, such “intensification” techniques provide a greater challenge to the musculoskeletal system and thus may enhance RT adaptations over and above that of traditional DCER protocols.

Eccentric actions, which involve forcible lengthening of the working muscles, allow the use of higher absolute loads compared to concentric actions. Although the mechanisms are not entirely clear, EO (i.e., performing eccentric actions with loads in excess of concentric training) has been shown to enhance acute anabolic signaling and satellite cell activation, as well as long-term neuromuscular adaptations compared to traditional DCER protocols^7^. However, safe and effective performance of EO requires either a spotter or specialized equipment (e.g., flywheel machinery, X-Force resistance machines, etc.) in many instances, thereby limiting its practical applicability.

VR training can take on several forms including the use of chains, bands, and pneumatic apparatus. One of the more novel applications of the concept involves attempting to match the applied resistance to human strength curves throughout a given range of motion during RT. Specifically, various machines have been designed to provide increased resistance in the joint angles where muscles can exert greater levels of torque and decreased resistance where muscles produce less torque^8^. This objective is primarily accomplished through implementation of cams and levers in commercial grade equipment. Theoretically, the use of these machines heightens mechanical tension to the working musculature and thus may optimize the adaptive training response. However, evidence remains equivocal as to whether such equipment effectively replicates human torque capabilities^9,10^, calling into question its utility. Moreover, because VR requires specialized equipment, it is impractical outside a gym setting.

Modern technology has facilitated the ability to make advanced training methods more accessible to the general public. A consumer-oriented unit called Tonal incorporates various advanced training features, including EO and VR, via the use of computerized electromagnetic resistance. However, no study to date has investigated the efficacy of these features in comparison to traditional training methods.

Surface electromyography (sEMG) is a popular research tool for investigating various aspects of muscle mechanics. Among its applications, sEMG can help to provide insights into neuromuscular behavior during exercise performance^11^, and thus potentially guide practical prescription. Indeed, some research indicates that sEMG amplitudes may be associated with group-based changes in muscle cross-sectional area^12^, although the veracity of this premise remains contentious^13^. Therefore, the purpose of this study was to compare the effects of electromagnetic resistance alone, as well as in combination with variable resistance or accentuated eccentric methods, with traditional DCER exercise on myoelectric activity during elbow flexion. We hypothesized that: (1) electromagnetic resistance would produce similar sEMG amplitude compared to DCER; (2) EO training would produce greater sEMG amplitude on the eccentric actions compared to the other conditions, and; (3) VR training would produce greater sEMG amplitude throughout the range of motion on the concentric actions compared to the other conditions.

## Methods

### Participants

Participants were 16 young, resistance-trained male and female volunteers (male = 10, female = 6; age 25.8 ± 5.5 yrs; height 172.3 ± 7.9 cms; weight 79.8 ± 14.6 kgs; training experience 6.7 ± 4.4 yrs) recruited as a convenience sample from the university campus. This sample was estimated by G*power based on an analysis of variance within-between repeated measures model using an α of 0.05, a β of 0.8, a relatively large effect size (ES) of 0.4, and a correlation among repeated measures of 0.5 for sEMG amplitude at a given joint angle, consistent with previous research^14^. The estimate provided an actual statistical power of 0.88.

Inclusion criteria required that participants: (1) were between the ages of 18 to 40; (2) could read and speak English; (3) answered “no” to all questions on a physical activity readiness questionnaire (PAR-Q), (4) did not suffer from a neurological disorder (e.g., multiple sclerosis, cerebral palsy, etc.), and; (5) had at least 1 year of resistance training experience (defined as performing resistance training consistently over this period for at least 2 days per week) with experience performing the biceps curl exercise. Those receiving care for any upper body injury at the time of the study or those with an amputation of a limb were excluded from participation.

The study employed a within-participant randomized, cross-over design whereby all participants performed each of the following conditions: Elbow flexion using a dumbbell (DB); elbow flexion using a commercial electromagnetic resistance device (ELECTRO) (Tonal Corporation, San Francisco, CA, USA), VR elbow flexion using a setting on the electromagnetic device that attempts to match the level of resistance to the human strength curve (25% of weight variation), and; EO elbow flexion using a setting on the electromagnetic device that increases the load by 50% on the eccentric portion of each repetition^15^. All exercises were performed unilaterally with the right arm. Consistent with previous research^16^, the conditions were randomized in a counterbalanced fashion using online software (www.randomizer.org.) to ensure that the order of performance did not unduly influence results.

Each participant was informed about the risks and benefits of the study and signed a written informed consent prior to participation. Approval for the study was obtained from the university Institutional Review Board at Lehman College; research was performed in accordance with the Declaration of Helsinki. All training and data collection was performed at the same site. The methods for this study were preregistered prior to recruitment at: osf.io/un5ym.

### Initial assessment

Prior to sEMG analysis, participants reported to the lab for anthropometric assessment and 10 repetition maximum (RM) testing. Participants were instructed to refrain from eating for at least 8 h prior to testing, eliminate alcohol consumption for 24 h, abstain from upper body resistance training for 48 h and avoid any type of strenuous physical exercise for 24 h.

Participants’ height was measured to the nearest 0.1 cm using a stadiometer. Weight was assessed to the nearest 0.1 kg on a calibrated scale, which also provided an estimate of body fat percentage (InBody 770; Biospace Co. Ltd., Seoul, Korea). Right arm girth was assessed at the midpoint between the lateral epicondyle of the humerus and the acromion process of the scapula with the participant seated and arm hanging relaxed at the side of the body.

10-RM elbow flexion testing was carried out in DB, ELECTRO, VR, and EO conditions. Each condition was separated by a 10-min rest period to allow for sufficient recovery from the previous test. The 10-RM testing was consistent with recognized guidelines as established by the National Strength and Conditioning Association^17^. In brief, participants performed a general warm-up prior to testing that consists of light cardiovascular exercise lasting approximately 5 to 10 min. Afterward, the 10-RM load was assessed for each condition, with a successful attempt considered as the ability to complete a 10th repetition but not an 11th repetition with proper form (defined as excursing from full elbow extension to full flexion). If the participant was able to perform more than 10 repetitions, we increased the load by 5 to 10%. We provided a rest interval of 3 min between trials. The loads determined during this session were used during assessment of sEMG amplitude.

### Experimental assessment

At least 48 h but not more than 1 week after 10RM testing, sEMG analysis was conducted on each participant using a Delsys EMG Trigno™ Wireless EMG systems (Delsys Corporation, Boston, MA, USA) connected to a PC running EMGworks® 4.7.9 software and sampling at 2000 Hz. The sEMG was synced to a Vicon motion capture system (Vicon Motion Systems Limited, Oxford, UK) using 6 Vicon VERO cameras at a sampling rate of 100 Hz. Reflective markers were placed on the lateral aspect of the acromion, the medial and lateral epicondyle of the elbows, and the styloid process of the radius and ulna. Participants wore a tank top or sports bra to facilitate data acquisition.

For the sEMG assessment, participants were prepared by lightly shaving and then abrading the skin with an alcohol swab in the desired areas of sensor attachment to ensure stable contact and low skin impedance. After preparation, wireless sEMG smart sensors were attached parallel to the fiber direction of the biceps brachii (BB), anterior deltoid (AD) and brachioradialis (BR). Electrode placement was made on the right arm of each participant. The BB electrode was placed on the line between the medial acromion and the fossa cubit at 1/3 from the fossa cubit and the AD electrode was placed at one finger width distal and anterior to the acromion. These methods are consistent with the recommendations of SENIAM (Surface Electromyography for Non-Invasive Assessment of Muscles)^18^. Given that SENIAM does not provide guidelines for the BR, we placed this electrode on the proximal forearm where the muscle becomes superficial, 4 cms from the cubital fossae as described by Bailey et al.^19^. After all electrodes were secured, a quality check was performed to ensure sEMG signal validity.

### Maximal voluntary isometric contraction

Maximal voluntary isometric contraction (MVIC) data was obtained for the desired muscles by performing a resisted isometric contraction as outlined by Hislop and Montgomery^20^. After an initial warm up consisting of 5 min of light cardiovascular exercise and slow dynamic stretching in all three cardinal planes, MVIC testing was carried out as follows: For the biceps brachii, participants sat upright with elbow flexed at 90° and forearm supinated. Resistance was applied at the wrist with the other hand cupping the elbow for support. Participants were asked to flex their right elbow by slowly increasing the force of the contraction so as to reach a maximum effort after approximately 3 s. Participants then held the maximal contraction against resistance for 3 s before slowly reducing force over a final period of 3 s. The same procedure was performed for the brachioradialis except with the forearm in neutral position. For the anterior deltoid, participants sat upright with the shoulder flexed at 90°, arm straight and forearm pronated. Resistance was applied at the distal humerus, just above the elbow, with the other cupping the shoulder for support. Participants were asked to flex the shoulder by slowly increasing the force of the contraction so as to reach a maximum effort after approximately 3 s. Participants then held the maximal contraction against resistance for 3 s before slowly reducing force over a final period of 3 s. A recovery period of 2 min was provided between trials.

### Exercise description

Ten minutes after MVIC testing, participants performed each of the elbow flexion conditions from full extension (0°) to as far as the participants elbow could flex with palm supinated throughout the movement. A 10-min rest period was provided between trials to ensure that fatigue did not confound results. To enhance ecological validity, we opted not to use a metronome to control tempo. Rather, participants were instructed to perform concentric actions in a controlled but forceful manner and to control eccentric actions by resisting gravity (cadence of ~ 2 s on each action). Sets were carried out to the point of momentary muscular failure—the inability to perform another concentric action with proper form. Verbal inducements were provided to each participant before and during performance by the research team to ensure that trials were carried out in the prescribed manner.

### Instrumentation and processing

The labeled marker data were imported in Matlab (Matlab 2019) to determine the elbow joint angle in the concentric and eccentric phase. To this purpose, a vector was defined from the lateral epicondyle of the elbow to the average position of the styloid process of the radius and ulna, and from the lateral epicondyle of the elbow to the marker on the lateral aspect of the acromion. The angle between the vectors was calculated and taken as the elbow angle. The concentric phase was defined as the time period from maximum to minimum joint angle, while the eccentric phase was defined as the period from minimum to maximum joint angle.

Raw sEMG signals were filtered by a 20–450 Hz zero-lag Butterworth bandpass filter, with a 2-pole low-Pass (40 dB/decade, or 2nd order) and a 5 pole High-Pass (80 dB/decade, or 5^th^ order). The filtered sEMG signals were then processed using a 100 ms moving window Root Mean Square (RMS) procedure, prior to normalizing against the respective maximum value taken from either the MVIC or during any of the trials. The max value was taken as the highest value over 500 samples. The resulting signal was then filtered using a 4th order Butterworth with a low pass cut-off at 2 Hz to further smooth the signal, and the amplitude at five joint angles (30°, 50°, 70°, 90°, and 110°) was determined for both concentric and eccentric actions. The second filter was applied to reduce the influence of naturally occurring fluctuations in the sEMG signal on the joint-angle specific EMG value.

### Statistical analysis

EMG amplitude was assessed under four conditions (DB, ELECTRO, VR, EO) at five joint angles (30°, 50°, 70°, 90°, 110°) for both concentric and eccentric actions. The preregistered analysis for this study intended to employ a frequentist approach with a focus on interpreting the results on a continuum using all statistical outcomes in combination with theory and practical considerations. Prior to any analysis of data collected, however, it was decided that a Bayesian approach better matched the overall intention, which was to explore potential differences in myoelectric activity and assess the extent to which they may be meaningful rather than dichotomize results. In addition, Bayesian analyses with their sampling procedures provide a relatively simple tool to model extensive repeated measures structure within data. In the interests of transparency, we conducted statistical analyses using both approaches and have presented the original methods and results from the preregistered approach in supplementary files (see Supplemental Table ST1).

The primary analysis comprising Bayesian mixed effects models were conducted separately for each muscle and phase of movement (concentric or eccentric), with data combined across joint angles and repetitions. Mixed effect models included: (1) fixed effects for condition, joint angle and repetition; (2) random effects for participant intercepts; and (3) an autoregressive ar(1) term to account for stronger associations between adjacent repetitions. Fixed effects for condition were set by including DB as the reference level such that a positive/negative coefficient indicated increased/reduced EMG activity for the comparator (ELECTRO, VR or EO). Inferences on population mean differences in EMG activity between the conditions were made using the posterior samples from fixed effects and interpreting median values and 95% credible intervals (95% CrI’s). Posterior samples were also used to calculate the probability that differences exceeded the pre-determined thresholds of 0, 5 and 10%MVIC to better interpret the practical significance of results. The secondary analysis was conducted using the same mixed effects models but separated across joint angles with results presented in the supplementary files (Table ST1). To visualize analyses, plots were created using the mean values with standard errors calculated through 1000 bootstrap samples with replacement and direct calculation of the standard deviation of the bootstrapped means.

Default priors were used for all parameters including improper flat priors (any value is considered equally likely), the LKJ(1)-correlation prior^21^ for the correlation matrix linked to participant intercepts, and half Student-t priors with 3 degrees of freedom for standard deviations ^22^. All models were fitted within the brms package that interfaced with the Bayesian software Stan^21^. Models were fitted with 5 chains each comprising 10,000 sets of posterior estimates. Convergence of parameter estimates were obtained for all models with Gelman-Rubin R-hat values below 1.1^22^.

## Results

**Figure 1:**
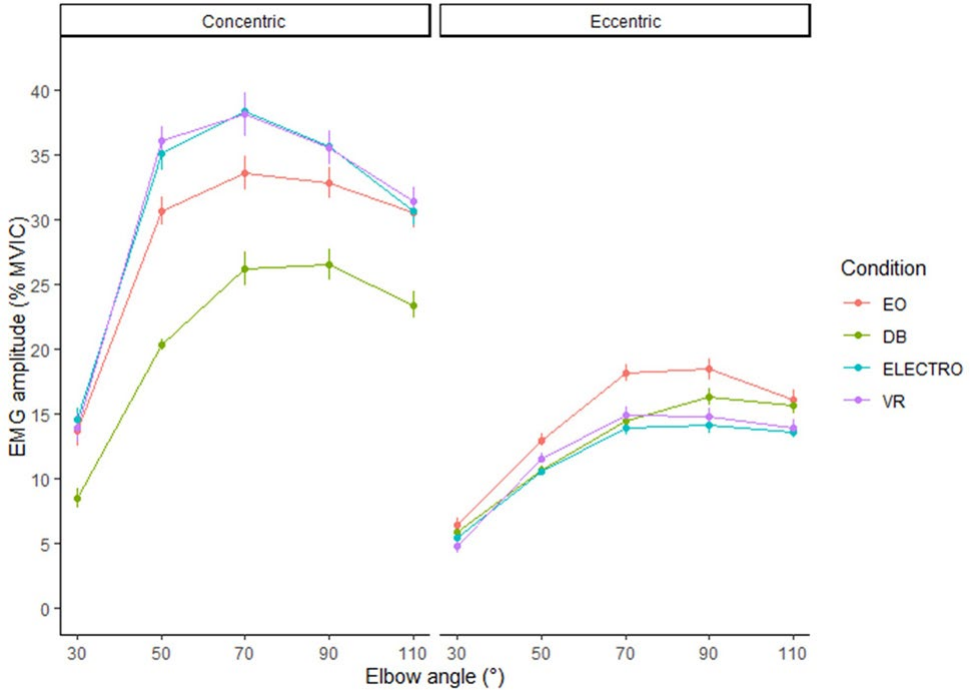
sEMG amplitudes for the deltoid presented across conditions and summarized across repetitions. Circles represent means and error bars represent ± one standard error calculated from bootstrap samples. *sEMG* Surface electromyography, *EO* Eccentric overload, *DB* Dumbbell, *ELECTRO* Electromagnetic resistance, *VR* Variable resistance.

**Figure 2:**
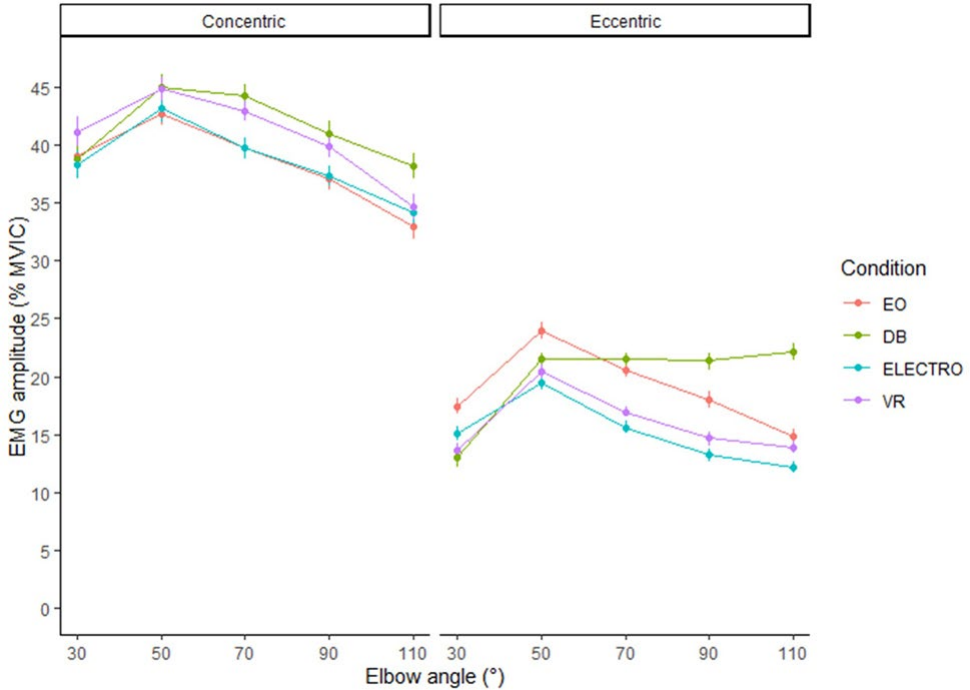
sEMG amplitudes for the biceps brachii presented across conditions and summarized across repetitions. Circles represent means and error bars represent ± one standard error calculated from bootstrap samples. *sEMG* Surface electromyography, *EO* Eccentric overload, *DB* Dumbbell, *ELECTRO* Electromagnetic resistance, *VR* Variable resistance.

**Figure 3:**
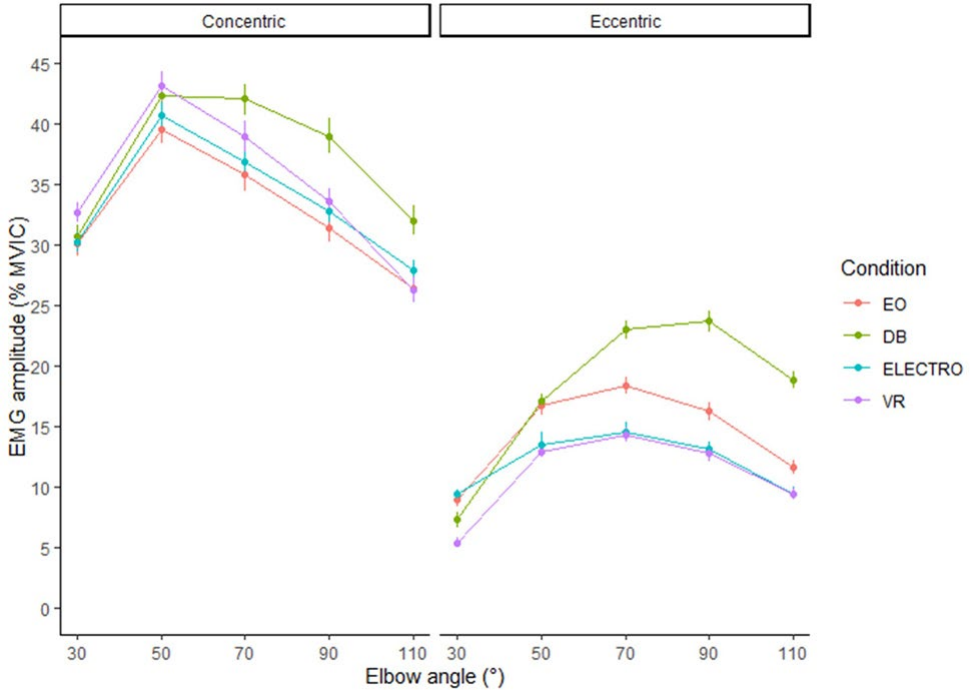
sEMG amplitudes for the brachioradialis presented across conditions and summarized across repetitions. Circles represent means and error bars represent ± one standard error calculated from bootstrap samples. *sEMG* Surface electromyography, *EO* Eccentric overload, *DB* Dumbbell, *ELECTRO* Electromagnetic resistance, *VR* Variable resistance.

Comparisons of sEMG amplitude across conditions for the deltoid, biceps and brachioradialis are presented in Figs. 1, 2 and 3, respectively.

Analysis across all plots shows greater %MVIC during the concentric phase with values influenced by joint angle. Increases followed by plateaus were identified for the deltoid as joint angles progressed from 30 to 110°, whereas inverted-V shapes were identified for the biceps and brachioradialis.

Results from the mixed effects autoregressive models comparing conditions are presented in Table 1. The largest differences between conditions were identified for the anterior deltoid, where median estimates indicated greater concentric sEMG amplitude (~ 7 to 10%) with EO, ELECTRO and VR compared with DB. The probability was high that differences exceeded 5% (*p* ≥ 0.952) but relatively low for exceeding 10% (*p* ≤ 0.407). Eccentric anterior deltoid sEMG amplitude was highest with EO but unlikely to exceed a 5% difference relative to DB (*p* < 0.001).

**Table 1:** Parameter estimates and probabilities from mixed effects autoregressive models describing differences between conditions.

Model	Eccentric overload relative to Dumbbell				Electromagnetic relative to Dumbbell				Variable Resistance relative to Dumbbell				Model parameters	
	Estimate and CrI	Probabilities			Estimate and CrI	Probabilities			Estimate and CrI	Probabilities			Ar(1)	${\sigma }_{ID}$
	Difference [95% CrI]	*p*(0%)	*p*(5%)	*p*(10%)	Difference [95% CrI]	*p*(0%)	*p*(5%)	*p*(10%)	Difference [95% CrI]	*p*(0%)	*p*(5%)	*p*(10%)	Estimate [75%CrI]	Estimate [75%CrI]
Deltoid														
Concentric	6.9 [4.7 to 9.1]	> 0.999	0.952	0.004	9.0 [6.7 to 11.3]	> 0.999	> 0.999	0.192	9.7 [7.4 to 12.0]	> 0.999	> 0.999	0.407	0.69 [0.67 to 0.71]	9.4 [7.5 to 11.9]
Eccentric	1.8 [0.7 to 2.8]	> 0.999	< 0.001	< 0.001	− 1.1 [− 2.1 to − 0.1]	0.978	< 0.001	< 0.001	− 0.7 [− 1.7 to 0.3]	0.898	< 0.001	< 0.001	0.67 [0.65 to 0.69]	5.2 [4.2 to 6.6]
Biceps														
Concentric	− 1.7 [− 4.4 to 1.0]	0.886	0.007	< 0.001	− 1.2 [− 3.9 to 1.6]	0.798	0.003	< 0.001	0.5 [− 2.1 to 3.3]	0.658	< 0.001	< 0.001	0.72 [0.70 to 0.74]	6.3 [5.0 to 8.1]
Eccentric	0.1 [− 1.6 to 1.7]	0.524	< 0.001	< 0.001	− 3.6 [− 5.2 to − 1.9]	> 0.999	0.044	< 0.001	− 3.2 [− 4.8 to − 1.5]	> 0.999	0.015	< 0.001	0.78 [0.76 to 0.79]	4.2 [3.4 to 5.4]
Brachio														
Concentric	− 3.2 [− 5.2 to − 1.3]	> 0.999	0.036	< 0.001	− 2.3 [− 4.2 to − 0.4]	0.993	0.003	< 0.001	− 1.2 [− 3.1 to 0.8]	0.885	< 0.001	< 0.001	0.65 [0.63 to 0.66]	10.2 [8.4 to 13.0]
Eccentric	− 2.8 [− 4.3 to − 1.1]	> 0.999	0.002	< 0.001	− 4.6 [− 6.2 to − 2.9]	> 0.999	0.321	< 0.001	− 6.2 [− 7.7 to − 4.6]	> 0.999	0.927	< 0.001	0.75 [0.73 to 0.76]	5.2 [4.2 to 6.5]

CrI: Credible interval. Ar(1): Autoregressive estimate. ${\sigma }_{ID}$: Sigma estimate describing standard deviation of participant intercepts. *p*(${\varvec{x}}$%), the probability that mean difference between dumbbell and specified condition exceeds ${\varvec{x}}$ (if median estimate is positive) or is less than $-{\varvec{x}}$ (if median estimate is negative).

Concentric biceps brachii sEMG amplitude was relatively similar between conditions, with any observed differences across joint angles likely trivial (Table 1). In contrast, evidence indicated a greater eccentric amplitude with DB compared to ELECTRO and VR (*p* > 0.999), but unlikely to exceed a 5% difference (*p* ≤ 0.044).

Evidence was observed for greater concentric (*p* ≥ 0.885) and eccentric (*p* ≥ 0.999) brachioradialis sEMG amplitudes with DB compared to all other conditions. In general, however, differences were unlikely to exceed 5% (Table 1). Analyses conducted across individual joint angles showed similar patterns to those described above but with limited differences at 30° (see Supplementary files).

## Discussion

This is the first study to compare sEMG amplitudes in traditional free weight exercise with EO and VR using electromagnetic technology. The results indicated clear evidence of differences in sEMG amplitude across multiple muscles and conditions during elbow flexion exercise. Where these differences were observed, however, magnitudes were generally modest between conditions (< 10%) and therefore unlikely to be practically meaningful. Consistent with our hypothesis, the electromagnetic technology produced similar sEMG amplitudes compared to free weights. Contrary to our initial hypothesis, however, the EO condition did not produce greater sEMG amplitude on the eccentric actions compared to the other conditions, and VR training did not produce greater sEMG amplitude on the concentric actions at any of the studied angles compared to the other conditions. What follows is a discussion of the specific findings and their potential practical implications for performance.

In general, sEMG amplitude changed across joint angles irrespective of condition. Consistent with previous research, sEMG amplitude was higher during concentric vs eccentric actions for all conditions^23^. Concentrically, amplitude for the biceps brachii and brachioradialis displayed an inverted ‘V’ shape, with amplitude peaking at ~ 50–70° and then declining thereafter (Figs. 2 and 3, respectively). Alternatively, amplitude for the anterior deltoid increased more severely during the initial 70° and then showed only a slight decline thereafter (Fig. 1). Eccentrically, the patterns generally were mirror images of the concentric action, with the exception of the biceps brachii in the DB curl, which maintained a constant amplitude from 110 to 50° before rapidly declining in the final 30° (Fig. 2). These findings provide insights into the divergent responses between both joint actions as well as muscles during elbow flexion exercise.

In regard to specific muscles, sEMG amplitude was modestly higher in all electromagnetic conditions compared to the DB for the anterior deltoid on the concentric action. This finding was observed across all joint angles and is consistent with previous research using a cable-based apparatus versus a selectorized machine for elbow flexion exercise^24^. The differences were most apparent in the ELECTRO and VR conditions, with results likely to exceed 10%. Eccentrically, amplitude for EO was modestly higher than other conditions, but likely of little practical significance. Results may be due in part to the positioning of participants during use of the electromagnetic device. Because the electromagnetic device used in this study is wall-mounted, participants had to be positioned perpendicularly to the unit with its attachment slightly posterior to participants so that the motion capture system could locate all markers throughout the range of motion of each exercise. We speculate that the backward pull of the cable in this configuration may have elicited a moment that necessitated the anterior deltoid to resist shoulder hyperextension in an effort to stabilize the upper arm at the torso during performance. It remains unclear if/how assuming different body positions vis-a-vis the electromagnetic device (e.g., facing the unit so that the attachment is in front of the participant) might affect muscle excitation to the anterior deltoid; this requires future study.

For the biceps brachii, sEMG amplitude was generally similar across conditions concentrically. Amplitude for the DB and VR were slightly higher than for ELECTRO and EO (< 5%), and unlikely of practical significance. DB and EO produced the highest amplitudes eccentrically, but the magnitude of differences between all conditions was likely trivial (< 5%). As mentioned above, the DB produced a distinct pattern whereby biceps brachii sEMG amplitude remained relatively constant during the initial lowering phase, and then sharply declined at 50°. A similar pattern of amplitude across joint angles has been reported previously during elbow flexion with a dumbbell^14^, lending support to the veracity of this finding. Overall, results suggest all conditions evoke similar muscle excitation to the biceps brachii throughout the range of motion on concentric actions. Discrepancies in amplitude between the DB and the electromagnetic device conditions on the initial phase of the eccentric action remain to be elucidated but conceivably may be due, at least in part, to kinetic differences between modalities. However, the summed eccentric amplitudes across training angles were relatively similar, thus calling into question any practical significance of this finding.

For the brachioradialis, the DB produced higher amplitudes both concentrically and eccentrically, with the greatest differences occurring between 50 and 110° of elbow flexion. Concentrically, the differences between conditions were likely of trivial consequence (< 5%). However, eccentrically amplitudes for the DB likely exceeded those of ELECTRO and VR by ~ 5% but < 10%. The findings suggest that the DB evokes slightly greater muscle excitation to the brachioradialis compared to the electromagnetic device conditions, more so during the eccentric actions. However, the magnitude of differences between conditions are relatively modest and of questionable practical significance.

Only a few previous studies have compared sEMG amplitude in EO versus traditional modes of training with combined concentric/eccentric actions. Sarto et al.^15^ reported that mean normalized integrated sEMG was ~ 30% higher in the vastus lateralis for EO with the eccentric action performed at 150% of concentric load versus traditional training at 70 to 80% 1RM. Similarly, Castro et al.^25^ demonstrated that EO (performed at 100% of 1RM eccentrically) elicited greater eccentric sEMG amplitudes for the pectoralis major and triceps brachii compared to traditional training in the bench press at both 30 and 80% 1RM. Although speculative, reasons for discrepancies between our findings and the aforementioned studies may be explained by differences in the manner in which EO was applied (i.e., weight releasers versus electromagnetic), type of exercise (i.e., multi- versus single-joint) and/or muscles analyzed.

In regard to VR, multiple studies have investigated amplitudes using bands and chains versus traditional training modalities^26–30^. Although such studies are of general interest, bands and chains alter kinetics by increasing resistance in an ascending fashion and thus results cannot be compared to the present study. A limited number of studies have compared myoelectrical activity in VR modalities that attempt to match resistance to the human strength curve with traditional isotonic exercise, with conflicting results. Peltonen et al.^31^ employed fine wire EMG analysis to compare myoelectrical activity of the glenohumeral muscles during external rotation using a cam-based VR versus a cable pulley device at 10%, 50% and 100% of the torque measured in participants’ 1RM. Results showed that VR tended to produce a more consistent amplitude across joint angles than the cable device, particularly in the 50% and 100% loading conditions. Vailas et al.^32^ used fine wire electrodes to assess EMG amplitude of the biceps brachii, triceps brachii, semimembranosus and vastus medialis during a single concentric action at 75% of 1RM in a cam-based VR machine versus free weights. Overall, EMG amplitude tended to be greater with free weights compared to VR. Results generally showed that free weights produced an ascending amplitude pattern from the start to finish position, except in the triceps brachii where the pattern was reversed. Conversely, VR produced a relatively constant amplitude across joint angles, except in the vastus medialis which displayed an ascending pattern. It should be noted that the specific exercises used to assess each muscle were poorly described, thereby limiting the ability to scrutinize findings.

The present study had several limitations that must be acknowledged. First, our findings are specific to a young, resistance-trained population and cannot necessarily be extrapolated to other populations including youth, untrained, and older individuals. Second, the advanced training methods investigated herein are specific to a computer algorithm applied under electromagnetic conditions. Thus, results cannot necessarily be extrapolated to other forms of variable resistance and eccentric overload. Third, the findings are specific to isolated elbow flexion exercise and thus cannot necessarily be generalized to multi-joint movements or exercises for other joints/muscles. Finally and importantly, although sEMG is frequently used to predict muscular adaptations over longitudinal resistance training programs, and some evidence suggests a potential association between sEMG amplitudes and changes in muscle cross-sectional area^12^, evidence supporting such a relationship remains inconsistent and equivocal^13^. Moreover, if sEMG can indeed predict such responses, research has yet to quantify the magnitude at which differences in sEMG amplitude between different conditions translates into meaningful differences in chronic improvements. Although we have attempted to draw practical implications based on a spectrum of percentage changes, our inferences remain speculative and require further research for confirmation.

## Conclusions

In conclusion, differences in sEMG amplitude were observed across conditions during isolated elbow flexion exercise. The electromagnetic device and its associated advanced training modes tended to produce greater amplitudes in the anterior deltoid, while DB tended to produce greater amplitudes in the brachioradialis; amplitude for the biceps brachii was relatively similar between conditions. Overall, any observed differences were relatively modest, equating to magnitudes of ~ 5% and not likely greater than 10%. These differences would seem to be of minimal practical significance.

Andersen et al.^33^ speculated that sEMG amplitudes should reach a minimum threshold of 40% MVIC to stimulate strength adaptations. The present study showed that each tested condition met or exceeded this threshold for the target muscle (biceps brachii) on the concentric action, suggesting all conditions provide a sufficient stimulus for strength improvements in this muscle. It should be noted that the hypothesis for the proposed threshold is based on the intensities of load employed in training studies, which may not reflect the actual relationship between sEMG and loading. Further research is needed to determine minimum thresholds for sEMG to produce chronic muscular adaptations via regimented RT.

Overall, the findings would seem to suggest that electromagnetic technology produces a similar muscle excitation to dumbbells during elbow flexion, and thus conceivably could be considered a viable alternative for RT programs in resistance-trained individuals. However, contrary to expectations, the advanced training methods associated with the electromagnetic device did not generally produce a heightened sEMG response. Although the intention of variable resistance training is to match the resistance to the human strength curve and thus enhance the stimulus throughout the range of motion, the VR tended to display similar amplitudes compared to other conditions. Similarly, while EO is intended to provide a greater stimulus during eccentric actions, this effect was generally not observed during performance compared to the other conditions in the target muscle. These results call into question the benefits of employing VR and EO with electromagnetic technology, at least from the standpoint of increasing muscle excitation to the working musculature. The implications of these findings to long-term muscular adaptations remain to be determined and require further investigation.

## Supplementary Information

Supplementary Information.

## Data Availability

Data is available at the Open Science Framework site where the study was preregistered: osf.io/un5ym.
