# Supplementary material for: Myoelectric activity during electromagnetic resistance alone and in combination with variable resistance or eccentric overload
Source: Sci Rep. 2023 May 22;13:8212. doi: 10.1038/s41598-023-35424-w (PMC10203319; doi:10.1038/s41598-023-35424-w)
Supplement: Supplementary file 1 — Supplementary Information. [file 41598_2023_35424_MOESM1_ESM.docx]

**Supplementary file X: Pre-registered statistical analysis.**

The following supplementary file includes details of the statistical methods and subsequent results of the original analysis approach presented in the pre-registration. We followed the statistical methods as described directly from the pre-registration: “The primary analysis will be based on a mixed effects model with fixed effect terms for joint angle and condition; subject will be treated as a random effect. The primary contrasts of interest are the 4 pairwise comparisons of conditions, averaged across repetitions. Hochberg’s modified Bonferroni procedure will be used to adjust for multiple comparisons. Effect sizes will be calculated using partial eta squared. 95% confidence intervals (CI) will be reported for all outcomes.”

**Deltoid**

The following table provides the means and standard deviations of EMG activity (%MVIC) averaged across joint angles for the four conditions.

| Mean ± Sd | Dumbbell | Eccentric overload | Electromagnetic | Variable resistance |
| --- | --- | --- | --- | --- |
| **Concentric** | 21.2 ± 14.2 | 28.8 ± 16.6 | 31.6 ± 16.8 | 31.6 ± 17.9 |
|  |  |  |  |  |
| **Eccentric** | 12.7 ± 7.8 | 14.7 ± 9.3 | 11.8 ± 7.1 | 12.2 ± 8.1 |

Pairwise comparisons:

The following table presents the pairwise %MVIC mean difference estimates and the adjusted p-values based on Hochberg’s modified Bonferroni procedure. Pairwise differences show columns relative to rows.

|  | Eccentric overload | Electromagnetic | Variable resistance |
| --- | --- | --- | --- |
| **Concentric** |  |  |  |
| Dumbbell | 7.5 [95%CI:4.2 to 10.7]  ***p*<0.001** | 9.8 [95%CI:6.6 to 13.0] ***p*<0.001** | 9.9 [95%CI:6.7 to 13.1]  ***p*<0.001** |
|  |  |  |  |
| Eccentric overload |  | 2.3 [95%CI:-0.9 to 5.5] *p=*0.323 | 2.4 [95%CI:-0.8 to 5.7]  *p=*0.427 |
|  |  |  |  |
| Electromagnetic |  |  | 0.1 [95%CI:-3.1 to 3.3] *p=*0.945 |
|  |  |  |  |
|  | Eccentric overload | Electromagnetic | Variable resistance |
| **Eccentric** |  |  |  |
| Dumbbell | 2.0 [95%CI:0.6 to 3.4] ***p*=0.017** | -1.0 [95%CI:-2.4 to 0.3] *p*=0.392 | -0.6 [95%CI:-2.0 to 0.7] *p*=0.719 |
|  |  |  |  |
| Eccentric overload |  | -3.0 [95%CI:-4.4 to -1.7] ***p*<0.001** | -2.6 [95%CI:-4.0 to -1.3] ***p*<0.001** |
|  |  |  |  |
| Electromagnetic |  |  | 0.4 [95%CI:-0.9 to 1.8] *p=*0.551 |

For concentric repetitions partial eta squared was equal to 0.14 for condition and 0.19 for joint angle. For eccentric repetitions actions partial eta squared was equal to 0.07 for condition and 0.35 for joint angle.

**Biceps**

The following table provides the means and standard deviations of EMG activity (%MVIC) averaged across joint angles for the four conditions.

| Mean ± Sd | Dumbbell | Eccentric overload | Electromagnetic | Variable resistance |
| --- | --- | --- | --- | --- |
| **Concentric** | 41.4 ± 14.6 | 38.2 ± 13.8 | 38.6 ± 13.6 | 40.7 ± 13.3 |
| **Eccentric** | 20.0 ± 9.1 | 19.0 ± 8.8 | 15.1 ± 7.2 | 16.0 ± 7.4 |

Pairwise comparisons:

The following table presents the pairwise %MVIC mean difference estimates and the adjusted p-values based on Hochberg’s modified Bonferroni procedure. Pairwise differences show columns relative to rows.

|  | Eccentric overload | Electromagnetic | Variable resistance |
| --- | --- | --- | --- |
| **Concentric** |  |  |  |
| Dumbbell | -3.4 [95%CI:-6.5 to -0.3]  *p*=0.196 | -2.9 [95%CI:-6.0 to 0.2] *p*=0.350 | -1.1 [95%CI:-4.2 to 2.0]  *p*=0.998 |
|  |  |  |  |
| Eccentric overload |  | 0.5 [95%CI:-2.6 to 3.6] *p=*0.738 | 2.3 [95%CI:-0.8 to 5.4]  *p=*0.569 |
|  |  |  |  |
| Electromagnetic |  |  | 1.8 [95%CI:-1.3 to 4.9] *p=*0.764 |
|  |  |  |  |
|  | Eccentric overload | Electromagnetic | Variable resistance |
| **Eccentric** |  |  |  |
| Dumbbell | -1.0 [95%CI:-2.9 to 0.8] *p*=0.527 | -4.7 [95%CI:-6.6 to -2.9] ***p*<0.001** | -4.0 [95%CI:-5.9 to -2.2] ***p*<0.001** |
|  |  |  |  |
| Eccentric overload |  | -3.7 [95%CI:-5.5 to -1.8] ***p*<0.001** | -3.0 [95%CI:-4.8 to -1.1] ***p*<0.001** |
|  |  |  |  |
| Electromagnetic |  |  | 0.7 [95%CI:-1.2 to 2.6] *p=*0.468 |

For concentric repetitions partial eta squared was equal to 0.02 for condition and 0.03 for joint angle. For eccentric repetitions actions partial eta squared was equal to 0.10 for condition and 0.01 for joint angle.

**Brachioradialis**

The following table provides the means and standard deviations of EMG activity (%MVIC) averaged across joint angles for the four conditions.

| Mean ± Sd | Dumbbell | Eccentric overload | Electromagnetic | Variable resistance |
| --- | --- | --- | --- | --- |
| **Concentric** | 37.3.4 ± 16.0 | 32.8 ± 15.0 | 33.9 ± 13.3 | 35.0 ± 14.7 |
| **Eccentric** | 18.2 ± 10.6 | 14.6 ± 9.2 | 12.1 ± 8.5 | 11.1 ± 7.1 |

Pairwise comparisons:

The following table presents the pairwise %MVIC mean difference estimates and the adjusted p-values based on Hochberg’s modified Bonferroni procedure. Pairwise differences show columns relative to rows.

|  | Eccentric overload | Electromagnetic | Variable resistance |
| --- | --- | --- | --- |
| **Concentric** |  |  |  |
| Dumbbell | -4.5 [95%CI:-7.3 to -1.8]  ***p*=0.008** | -3.1 [95%CI:-5.9 to -0.4] *p*=0.128 | -2.4 [95%CI:-5.1 to 0.4]  *p*=0.361 |
|  |  |  |  |
| Eccentric overload |  | 1.4 [95%CI:-1.4 to 4.2] *p=*0.640 | 2.2 [95%CI:-0.6 to 4.9]  *p=*0.376 |
|  |  |  |  |
| Electromagnetic |  |  | 0.8 [95%CI:-2.0 to 3.5] *p=*0.588 |
|  | Eccentric overload | Electromagnetic | Variable resistance |
| **Eccentric** |  |  |  |
| Dumbbell | -3.7 [95%CI:-5.7 to -1.6] ***p*=0.002** | -5.9 [95%CI:-8.0 to -3.9] ***p*<0.001** | -7.0 [95%CI:-9.1 to -5.0] ***p*<0.001** |
|  |  |  |  |
| Eccentric overload |  | -2.3 [95%CI:-4.3 to -0.254] *p*=0.056 | -3.4 [95%CI:-5.4 to -1.3] ***p*=0.004** |
|  |  |  |  |
| Electromagnetic |  |  | -1.1 [95%CI:-3.1 to 0.9] *p=*0.297 |

For concentric repetitions partial eta squared was equal to 0.04 for condition and 0.05 for joint angle. For eccentric repetitions actions partial eta squared was equal to 0.15 for condition and 0.06 for joint angle.
